# Hamstring Strain Injury (HSI) Prevention in Professional and Semi-Professional Football Teams: A Systematic Review and Meta-Analysis

**DOI:** 10.3390/ijerph18168272

**Published:** 2021-08-04

**Authors:** Carlo Biz, Pietro Nicoletti, Giovanni Baldin, Nicola Luigi Bragazzi, Alberto Crimì, Pietro Ruggieri

**Affiliations:** 1 Orthopedics and Orthopedic Oncology, Department of Surgery, Oncology and Gastroenterology (DiSCOG), University of Padua, 35128 Padua, Italy; pietrosea@gmail.com (P.N.); giovannibaldin@gmail.com (G.B.); albe.crim@gmail.com (A.C.); pietro.ruggieri@unipd.it (P.R.); 2 Department of Neurosciences, Institute of Human Anatomy, University of Padua, 35128 Padua, Italy; 3 Department of Mathematics and Statistics, Laboratory for Industrial and Applied Mathematics (LIAM), York University, Toronto, ON M3J 1P3, Canada; robertobragazzi@gmail.com

**Keywords:** hamstring, injuries, prevention, football, professional

## Abstract

Hamstring Strain Injuries (HSIs) are the most common type of lesion in professional footballers and the leading cause of absence days from sports. However, recent studies have shown that high-level football teams apparently do not apply any HSI prevention protocol. The aim of the study was to determine the effect of preventive strategies and protocols in reducing the incidence of hamstring muscle injuries in professional and semi-professional football teams. A literature search of PubMed/MEDLINE, ISI/Web of Science and Scopus databases was conducted with the keywords “hamstring* and (injury* or strain) and prevent* and (soccer or football)”. Quality and bias assessment was completed through the Kennelly modified scale. The Injury Incidence Rate (IIR) and the Incidence Rate Ratio (IRR) were assessed in the statistical analysis. In the meta-analysis, data were extracted, pooled and analysed with “Comprehensive Meta-Analysis Version 3.3.070” software. In total, 8 of the 1017 original search studies met the inclusion criteria of this review. The total exposure of the studies was 170,221.8 h, while the number of HSIs recorded was 165 in the intervention groups and 224 in the control groups. The average score of the quality assessment was 23.6/34. The meta-analysis of six of the eight included studies provided strong evidence that interventions are effective in reducing hamstring injuries. The IRR of the effect size was 0.443, with *p*-value = 0.001. The studies analysed applied different preventive strategies: the Nordic hamstring exercise, the FIFA 11+ programme and exercises for core stability or balance training. All these interventions proved to have a successful effect on prevention of hamstring injuries.

## 1. Introduction

Hamstring Strain Injuries (HSIs) are generally described as an elongation contracture, deep stretch or, more rarely, a tear of the muscles of the posterior compartment of the thigh, including, from lateral to medial: the biceps femoris, the semitendinosus and the semimembranosus [1,2,3]. These muscles cross two joints, the hip and the knee, and have a central role in the gait cycle during walking and running. These muscles are both hip extensors and knee flexors, and limit knee extension during the heel strike [4,5,6,7]. Among these muscles, the biceps femoris is the most injured during sports, followed by the semimembranosus and semitendinosus [8,9,10,11]. HSIs can occur during sharp turns or cutting in ball sports, or when running at full speed in sprinting [12,13,14]. They are the most frequent type of muscular injuries in professional football teams, where they cause more absence days than any other accident during the sporting season (25% of all absences due to injuries), with an average of 9 days of recovery time [11,15,16,17]. In professional football players, the incidence of injury varies between 1.06/1000 h (hours) and 5.87/1000 h of exposure to training and competition [11,18,19,20].

To better understand the impact of HSIs on football teams, between 2001 and 2014, Ekstrand et al. recorded 1614 HSIs in 36 teams from 12 European countries over 13 consecutive seasons [19,21]. Among those injuries, about one-third occurred during training, and the remaining occurred during official matches. In each season, 21.8% of the players had at least one injury [19]. The trend of HSIs was constant overall during the 13 seasons, although the HSIs suffered during training sessions increased by about 4% [21]. 

HSIs are usually caused by repeated eccentric muscular contractions [22,23,24,25]. They are very demanding in terms of energy and muscular tension due to the number of motor units recruited, with high mechanical and metabolic distress [24,26] and, consequently, more frequent Delayed Onset Muscle Soreness (DOMS) and acute or subacute tendinous lesions [27,28,29]. The relationship between eccentric muscular contraction and presentation of HSIs seems to be confirmed by the lower occurrence of these muscular lesions in swimming and cycling, two sports that are based on concentric contraction of the hamstrings [30,31]. On the contrary, football, rugby and basketball are the sports with higher occurrence of HSIs due to the amount of eccentric muscular contraction and wrong management, i.e., insufficient athletic preparation or athletes returning to sports before a complete recovery after an injury [23,32,33,34]. Eccentric strengthening is considered effective in the prevention of HSIs since they are associated with an eccentric strength deficit of the hamstrings and weakness during concentric action of the hip extensors in elite sprinters [35,36,37,38].

As already mentioned, football, rugby and basketball are the sports with higher incidences of HSIs. This is also because a muscle chronically damaged by indirect trauma is more susceptible to contractures and muscular straining [39]. However, many elements play a role in HSIs. These can generally be divided into intrinsic factors (related to anatomy, biology, biomechanics, the athlete’s technique) and extrinsic ones related to the athlete’s environment (inadequate warm-up, fatigue related to training, fitness level and training modalities) [23,40,41,42,43,44]. These factors can also be divided into modifiable (weight, muscular strength, muscular length and flexibility, resting time) and nonmodifiable (age, previous HSI, anatomy of the muscle) factors. 

These aspects highlight the importance of preventive treatments to reduce the occurrence of these injuries in football players [1,30,45,46,47,48]. Bahr et al. [14], analysing the adoption and implementation of Nordic Hamstring Exercise (NHE) by UEFA Champions League (UFC) teams during the 2012–2013 and 2013–2014 seasons, showed that HSI prevention protocols were not used or only partially used, and thus were ineffective. However, due to the high occurrence rate of HSIs and their many risk factors, professional football teams should have dedicated physiotherapy protocols to prevent them [1,21,49]. Since the management of these lesions is usually nonoperative treatment with surgery reserved for complete ruptures, the main focus should be on the prevention of HSIs to minimise injury occurrence and, therefore, absence days. In the case of key players, absence days can determine the success of a football team during a season.

Those considerations suggest the importance of identifying a preventive protocol for HSIs. However, despite the importance of such a topic, to the best of our knowledge, there are no systematic reviews and meta-analyses addressing the efficacy of preventive interventions targeting HSIs among professionals and semi-professional football players. Therefore, this systematic review is aimed to fill this gap of knowledge. More specifically, this study first aims to analyse and appraise the current literature describing the physiotherapy protocols and specific exercises for the prevention of HSIs in professional and semi-professional football players, and second to determine their true efficacy, highlighting the possible superiority of some over others. 

## 2. Materials and Methods

### 2.1. Study Reporting

The present systematic review and meta-analysis was conducted using the Preferred Reporting Items for Systematic Reviews and Meta-Analyses (PRISMA) guidelines, which were used to monitor all steps of the research [50]. The study protocol has been registered within the Open Science Framework depository (Identifier code: DOI 10.17605/OSF.IO/4DMRT).

### 2.2. Search Strategy

A medical librarian-assisted electronic comprehensive literature search was conducted in January 2021 using three different databases: PubMed/MEDLINE, ISI/Web of Science (WoS) and Scopus. In each database, an advanced search was conducted using a string of keywords which included ‘hamstring* AND (injury* OR strain) AND prevent* AND (soccer OR football)’, analysing all articles between 1 January 2009 and 31 December 2020. 

### 2.3. Selection Criteria

A full 12-year time selection was used, and only articles written in English and registered as Original Articles were included. Only randomised clinical trials, cohort studies, case-control studies and case series were selected if they met the following PICOS criteria:

- POPULATION: professional and semi-professional male and female footballers (only 11-a-side footballers were selected in the study, not including Futsal and Gaelic Football). Amateurs were excluded due to lack of a dedicated physiotherapy staff on their football teams and the lower amount of training and playing compared to their professional counterparts.
- INTERVENTIONS: application of conservative preventive protocols, including specific training (using Static-Dynamic Stretching, targeted exercises such as the Nordic hamstring exercise or Russian hamstring curls, core stability exercises, balance training exercises, etc.), physical therapy or pre-established protocols such as the FIFA 11+ programme, which was aimed at reducing the risk of HSI.
- COMPARISONS: articles that provided data that could make possible a general comparison among the different protocols adopted and with an acceptable number of hours of exposition to training and competitions.
- OUTCOMES: studies that had at least 2 months of follow-up, monitoring players between training and competition, and that included the number of hamstring muscle injuries or the number of injuries per 1000 h (HI/1000 h) of exposure, including training and official competitions.

### 2.4. Exclusion Criteria

All articles that did not analyse 11-a-side football, that did not study professional and semi-professional football players, that included post-operated athletes and that were not written in English were excluded. Case reports, interviews, opinions, single commentaries, narrative reviews, systematic reviews and meta-analyses were also excluded. The latter study designs were only checked to ensure that all relevant eligible articles were included. Articles with less than 2 months follow-up or with too few total hours of exposure between training and competition were excluded. Amateur footballers were not included because the risk of injury in amateur teams is much lower than in professional clubs, which would considerably increase the risk of bias in the statistical analysis.

### 2.5. Selection Method

The selection was based on the title and/or abstract’s content. If inclusion or exclusion of the article was not possible based on the title and/or abstract, the full-text article was retrieved and read. A cross-reference search of the selected articles was also performed to obtain other relevant articles for the study. Finally, the selected articles and references were reviewed and assessed independently by two authors (C.B. and P.N.) and then discussed together. Data extraction was completed independently by three assessors (G.B., N.B. and A.C.). If there was disagreement among the investigators regarding the inclusion or exclusion criteria, then the senior investigator made the final decision. The level of agreement was high, with a kappa statistic > 0.80 [51,52].

### 2.6. Quality Assessment

Given the heterogeneity among the studies in terms of study design and methodology adopted, the rating scale developed by Kennelly [53] was used to critically appraise the quality of the studies selected and retained in the present systematic review. This scale was elaborated starting from already existing tools: the Downs and Black scale [54] and the Physiotherapy Evidence Database (PEDro) scale [55]. We added an additional item to the 33 items of the Kennelly scale, which was chosen to establish whether the football players enrolled in the different studies had suffered from previous injuries [56]. Moreover, to account for the various types of study analysed (randomized and nonrandomized studies, including case series), 3 out of 34 items were phrased in two different forms. The quality of the studies was established as follows: poor ≤ 14; fair 15–19 and good ≥ 20 [57].

### 2.7. Risk of Bias

To evaluate the risk of bias, we selected 10 criteria directly from the items of the quality assessment, according to Kennelly et al. [53], which define the possible risk of bias as high, medium or low. The higher the score obtained by the study on the 10 items, the lower the risk of bias. With a score ≥ 9/10, the risk was considered low; with a score between 7/9 and 8/9, the risk was medium; and with a score ≤ 6/9, the risk was considered high.

### 2.8. Statistical Analysis

The outcomes analysed were the number of Hamstring Injuries (HI) recorded, the time of exposure to training and competition (where present), and the number of HI per 1000 h of exposure. From the collection of these data, the Injury Incidence Rate (IIR) was calculated for each sample using the following formula: (injury incidence/total exposure in hours) × 1000. This value represents players’ risk of injury. Consequently, the higher the IIR, the greater the risk for players to get injured. In addition, the Incidence Rate Ratio (IRR) was calculated (IIR^INTERVENTION^/IIR^CONTROL^). If the value of the IRR < 1, then the intervention had a preventive role against the risk of injury. If the IRR > 1, then the intervention was injurious to the players. The lower the IRR value, the more effective the preventive treatment.

The meta-analysis of the data was conducted using “Comprehensive Meta-Analysis Version 3.3.070” (CMA) software (Biostat, Englewood, NJ, USA). In the meta-analysis, data from the studies included in the review were analysed and pooled. The software calculated the natural logarithm of the IRRs of the various studies, the standard error and the variance. The sample size and the confidence interval (CI = 95%) were always considered in the analyses. Publication bias in terms of heterogeneity among studies was assessed by visually inspecting the funnel plot to evaluate the asymmetry of the generated graph. The results were considered statistically significant with a *p*-value < 0.05.

## 3. Results

### 3.1. Literature Search

**Figure 1:**
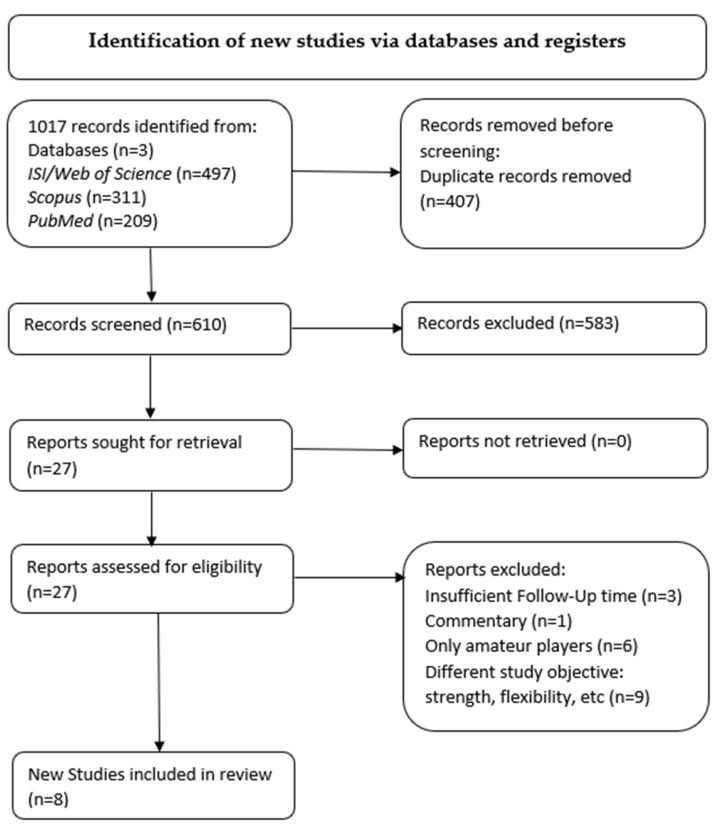
Preferred Reporting Items for Systematic Reviews and Meta-Analyses (PRISMA) flowchart representing the process for inclusion and exclusion of papers. For this study, 27 articles were assessed for eligibility after screening. Among these, 8 new studies were included in the analysis.

In the literature search, we found 497 articles from ISI/Web of Science, 311 from Scopus and 209 from PubMed/MEDLINE, for a total of 1017 articles. After removing 407 duplicates, 610 articles were included. In total, 206 titles were excluded. Of the 404 remaining abstracts, 27 full texts were included for review. Only 8 of these met the inclusion criteria and were analysed in the review (Figure 1) for a total of 3665 athletes, divided into 3458 males and 197 females. The eight studies consisted of five randomised clinical trials, two cohort studies and one case series. The randomised clinical trials were assigned a level of evidence I in Table 1. The cohort studies were assigned a level of evidence III, and the case series were assigned a level of evidence IV.

### 3.2. Patient Population

A total of 3665 football players was included in the review. Among them, 197 were female (5.38%). The group-weighted mean age in each study was 22.17 years, and the mean was 21.94 years, with a range of 20–24.8 years (Table 1).

### 3.3. Quality Assessment

In the quality assessment (Table 2), the average score of the 34 items was 23.6/34 points, with scores ranging from 13/34 to 30/34. The average quality percentage was 67.4%. In order from lowest to highest quality score: Melegati et al. [58], 13/34 points (38.2%) (poor quality); Kraemer et al. [59], 18/34 (52.9%) (fair quality); Grooms et al. [60], 21/34 (61.8%) (good quality); Whalan et al. [61], 25/34 (73.5%) (good quality); Elerian et al. [62] and Silvers-Granelli et al. [63], 26/34 (76.5%) (good quality); Petersen et al. [64], 28/34 (82.4%) (good quality); and Espinosa et al. [65], 30/34 (88.2%) (good quality). The average score tended to be medium-high, meaning that the average level of quality of the included studies was very satisfactory.

### 3.4. Risk of Bias

In the assessment of the risk of bias, the mean score was 7.75/10, corresponding to a medium risk of bias. A low risk was found in two studies (Whalan et al. [61], 9/10; Espinosa et al. [65], 9/10), a medium risk in five studies (Elerian et al. [62], 8/10; Silvers-Granelli et al. [63], 8/10, Grooms et al. [56], 8/10; Kraemer et al. [59], 8/10; Petersen et al. [64], 7/10) and a high risk in one study (Melegati et al. [58], 5/10).

### 3.5. Prevention Protocols

All studies adopted protocols, including exercises performed by the soccer players. No study mentioned physical therapies (TENS, Tecar, laser) or manual therapy techniques associated with the prevention protocols.

The standard FIFA 11+ programme was applied for 1107 players (Whalan et al. [61], Silvers-Granelli et al. [63], Grooms et al. [60]). FIFA 11+ is a programme divided into three parts for each session. The programme is designed for players and referees with the aim of reducing the incidence of lower limb injuries.

In total, 408 players (Whalan et al. [61]) performed the modified FIFA 11+, in which “Part 2” of the programme is performed at the end of training, as opposed to “Part 1”and “Part 3,” which are performed at the beginning of the session.

A total of 517 players (Elerian et al. [62], Espinosa et al. [65], Petersen et al. [64]) performed eccentric exercise as a preventive strategy. Among them, 495 players had this session before training, while 22 players had sessions both before and after training, with a greater focus on the Nordic hamstring exercise (NHE).

Kraemer et al. [59] analysed 5 intervention groups (the same team composed of 24 players in 5 different periods for a total of 120 players) in which balance training was performed. They calculated the total time of exercise executed by each of the players expressed in minutes. In the study by Melegati et al., 36 football players [58] performed core stability exercises, such as the prone abdominal body bridge, supine extension bridge, side bridge and push-up with trunk rotation. Lastly, in the study by Espinosa et al., 21 players [65] carried out global lower limb-strengthening exercises, such as *leg swings (FLS), side leg swings (SLS) and multiple jumping (MJ)* (Table 3).

No preventive programme was administered to 1456 football players, and the normal training sessions and schedules continued normally.

All articles provided the follow-up period, ranging from 12 weeks to 80 weeks. Apart from the studies by Espinosa et al. [65] and Petersen et al. [64], all studies explicitly stated the exposure time of the players to training and competition during the follow-up period expressed in h. The total time recorded was 170,221.8 h, of which 95,093.4 h was recorded for the intervention groups and 75,128.4 h for the control groups. A total average of 63.52 h was recorded per player, of which 72.76 h per player was recorded in the intervention groups and an average of 54.72 h per player was recorded in the control groups.

### 3.6. Outcomes

To calculate the Injury Incidence Rate, the following formula was used: (injury incidence/total exposure in hours) × 1000.

- Whalan et al. [61]: 1.9569 (IIR^CONTROL^), 1.5767 (IIR^INTERVENTION^)
- Elerian et al. [62]: 4.884 (IIR^CONTROL^), 0.5007 (IIR^INTERVENTION 1^),
- 2.0008 (IIR^INTERVENTION 2^)
- Espinosa et al. [65]: U/D (Undetermined)
- Kraemer et al. [59]: 12.4378 (IIR^CONTROL^), 8.9623 (IIR^INTERVENTION ^1), 4.99 (IIR^INTERVENTION 2^), 9.7629 (IIR^INTERVENTION 3^), 7.4349 (IIR^INTERVENTION 4^), 5.238 (IIR^INTERVENTION 5^)
- Melegati et al. [58]: 2.269 (IIR^CONTROL^), 1.369 (IIR^INTERVENTION^)
- Silvers-Granelli et al. [63]: 1.5613 (IIR^CONTROL^), 0.3619 (IIR^INTERVENTION^)
- Grooms et al. [60]: 2.0678 (IIR^CONTROL^), 0.37 (IIR^INTERVENTION^)
- Petersen et al. [64]: U/D

From these values, the following formula was used to calculate the IRR: IIR^INTERVENTION^/IIR^CONTROL^.

The IRR values were: 0.8057 (Whalan et al. [61]); 0.1025 for IG (intervention group) 1 and 0.4097 for IG2 (Elerian et al. [62]); U/D (Espinosa et al. [65]); 0.7206 for IG 1, 0.4012 for interv. 2, 0.7849 for IG3, 0.5978 for IG4, 0.4211 for IG5 (Kraemer et al. [59]); 0.6029 (Melegati et al. [58]); 0.2318 (Silvers-Granelli et al. [63]); 0.1789 (Grooms et al. [60]); U/D (Petersen et al. [64]).

### 3.7. Meta-Analysis

**Figure 2:**
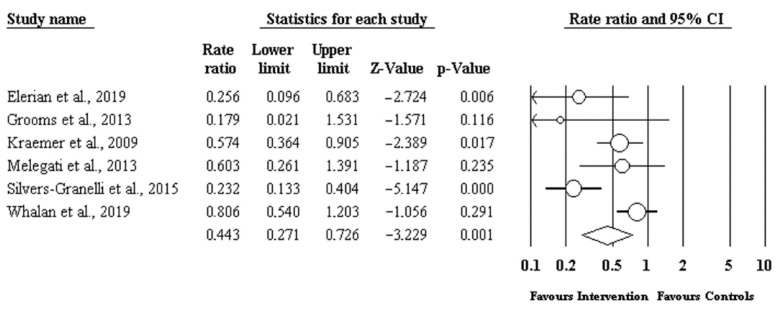
The forest plot visually depicts the effectiveness of the interventions. The preventive interventions proposed by the different articles analysed were effective in preventing HSIs with statistical significance (*p* = 0.001) among professional and semi-professional football players.

**Figure 3:**
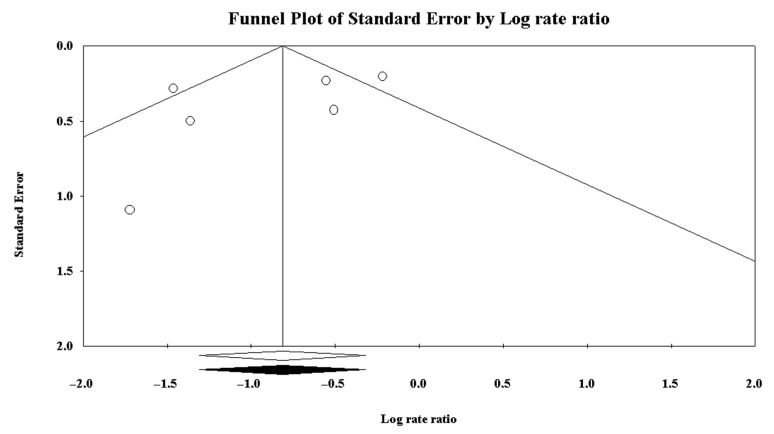
The funnel plot did not identify heterogeneity among the studies analysed. The x-axis Scheme 0. (95%CI 0.282–0.968), *p* = 0.039, whereas it was 0.379 for the randomized controlled trials (95%CI 0.149–0.968), *p* = 0.042.

Using the software “Comprehensive Meta-Analysis Version 3.3.070”53, a meta-analysis of the effectiveness of the prevention measures was carried out. We included six articles out of the eight in our review in the analysis, i.e., those in which it was possible to calculate the IRR. The forest plot in Figure 2 visually depicts the effectiveness of the interventions studied. The IRR of the effect size was 0.443 with *p*-value = 0.001. Figure 3 shows the funnel plot, where the natural logarithm of the Rate Ratio is depicted on the x-axis and the standard error of the studies is depicted on the y-axis. No element of heterogeneity among the samples analysed in the meta-analysis was identified.

At the meta-regression, there was no impact of study quality in terms of risk of bias on ES (coefficient—0.0067, standard error 0.0088 (95%CI—0.0239 to 0.0105), z-value= −0.77, *p* = 0.4442).

## 4. Discussion

HSIs are very common in athletes, both professional and semi-professional [17,33,42,66]. One of the most affected sports is football, where a high training load over time tends to overstretch the muscle group, predisposing athletes to a higher risk of injury [33,49,67,68,69]. Due to the high frequency of these injuries, it is very important to intervene with appropriate prevention strategies. Although HSIs are common injuries in footballers, there is a shortage of trials in the literature that consider the prevention of HSIs or compare the different prevention protocols available [10]. This review presents a comprehensive insight on the most up-to-date body of evidence in support of the effectiveness of preventive intervention protocols to reduce the incidence of HSIs in football players, examining the possible superiority of some over others.

As shown in the forest plot from Figure 2, the most important finding of this literature review was that, overall, the interventions proposed by the different articles analysed were beneficial in preventing HSIs. The specific effect size computed determines the strength of the relationship of HISs occurrence between the groups that underwent some prevention protocol and the control groups. More specifically, in our analysis, we found that the use of a prevention protocol had a beneficial effect on preventing HSIs as supported by the effect size of 0.443. The funnel plot shows that there was no publication bias or heterogeneity altering the final effect. However, it was not possible to identify the most effective prevention strategy due to the lack of literature on the topic and the insufficiency of sample numbers reported to allow a statistically significant comparison of the various interventions.

All the interventions included in this review reduced the IIR between the intervention and control groups (*p* = 0.001). No intervention had more HSIs than the controls for the same number of hours of exposure.

In our analysis, Whalan et al. [61] obtained the highest IRR (0.8057) and, consequently, the lowest percentage of efficacy between the intervention and control group. However, this result can be explained because, in the study by Whalan et al. [61], the control group was not prevention-free, but carried out the standard FIFA 11+. The only other study without a prevention-free control group was the one by Espinosa et al. [65]. However, due to missing information in the study about the exposure time to competitions and training, it was not possible to calculate the IRR. Therefore, it was included in the systematic review but not in the meta-analysis.

Since the statistical comparison was made between two different interventions [61], it did not allow an assessment of the absolute effectiveness of the FIFA 11+ protocol, which would have required a control group with no intervention. Instead, we compared the effectiveness of the two different versions of the FIFA 11+ protocol: the standard and the modified FIFA 11+ protocol.

The decrease of 19.43% between the IIR of the intervention group and the control group shows the highest effectiveness of the modified FIFA 11+ programme, although the differences did not achieve statistical significance (*p* = 0.291). The modified FIFA 11+ programme consists of performing “Part 2” of the programme at the end of training, separating it from the low- and high-intensity aerobic exercises, “Part 1” and “Part 3,” which are performed at the beginning of training. Hence, performing the strengthening exercises of the FIFA 11+ programme at the end of training is likely to reduce the risk of incidence of hamstring injury compared to the standard version of the programme [61,65].

The control groups in both studies by Silvers-Granelli et al. [63] and Grooms et al. [60] (the other two studies measuring the possible effectiveness of *FIFA 11+*) were prevention-free: the IRR values were the lowest among those recorded in the present review with rates of 0.23 (*p* = 0.000) and 0.18 (*p* = 0.116), respectively. Although presenting a higher IRR, and therefore appearing less beneficial, the study conducted by Silvers-Granelli et al. had a much greater impact on the overall effect size, since the sample had a total exposure time about 15.5-times higher than that of Grooms et al. [60]. However, both studies showed a very satisfactory effectiveness of the FIFA 11+ protocol. In the study by Elerian et al. study [62], IRR achieved a rate of 0.256, but two different interventions were examined and compared in the study: Intervention Group 1 had the lowest IRR of all interventions at a computed rate of 0.1025. Both groups performing the eccentric exercise and Nordic hamstring exercise (NHE) had fewer injuries than the control group. However, Intervention Group 1 (IG1), which performed the NHE both before and after training, had an IRR four-times lower than Intervention Group 2 (IG2), which only performed the NHE at the start of the sessions. This finding does not necessarily imply that performing the NHE at the end of training is more effective. Comparing the intervention exposure between the two groups, IG1 had, on average, twice as many NHE sessions as IG2. Therefore, making a comparison between the two groups statistically unreliable.

Although the IRR value is very low (IRR: 0.1025), in the meta-analysis presented in Figure 3, the study by Elerian et al. [62] showed a low significance on the final effect size (*p* = 0.006) due to the limited sample size. Kraemer et al. [59] monitored five balance training (BT) interventions, all performed by the same 24 players, and showed that the second BT intervention had the lowest IRR (0.4012). It also had the highest time in minutes of BT performed by the players (1080 min in total), reducing injuries by 59.88% compared to the control group. The third intervention, which included the lowest time in minutes of BT performed (450 min), achieved an IRR value of 0.7849, the highest in the study, reducing hamstring injuries by 21.51%. Among the five interventions in the study, the third intervention was the least effective.

Given the modest impact on the final effect size, and the inverse proportionality between the time in minutes of BT carried out by athletes and the Rate Ratio, BT appears to have an effective preventative role in reducing HSIs (*p* = 0.017).

The study by Melegati et al. [58] divided the football players into an intervention group that performed core stability exercises and a control group that performed global strengthening exercises for the lower limbs. In the intervention group, the IIR was reduced by 39.71% compared to the control group. Training to achieve better control of core stability seems to have a greater effect in preventing HSIs than non-selective, non-specific lower limb strengthening. This does not imply that muscle strengthening is ineffective as a form of prevention, but in the study, no effective criteria for training periodization were defined, so it was not possible to analyse the exercises performed by the control group. In addition, no general physical information on the sample was provided in this study, which prevents comparison with other studies in terms of parameters such as age and weight.

Both the studies by Espinosa et al. [65] and Petersen et al. [64] were not included in the meta-analysis due to the lack of exposure time of the players to competitions and training, which did not allow the calculation of the IIR of the professional group and consequently the IRR. Although they were not included, it is remarkable how the number of injuries in the intervention groups that performed *NHE* was reduced by 80% in the study by Espinosa et al., and by 71.15% in the study by Petersen et al. (the latter with a total sample size almost 22-times larger than the Spanish study).

The groups evaluated in the study by Petersen et al. [64] consisted of both professional and amateur footballers, and the authors did not specify which players from the two populations had injuries. As explained by these authors [44], amateurs play at lower intensities compared to professionals, both in competition and in training, and have, on average, approximately 1–2 fewer training sessions per week, increasing the risk of bias and possible heterogeneity in the study population.

In competitive sports, muscular and tendinous lesions are a very important element in the economic management of clubs due to the long recovery time [41,67,70]. However, in the studies analysed, the economic aspects were not directly investigated. It can be anticipated that strategies to prevent injuries to players would have an indirect, positive effect on the amount of money used to treat or replace injured players.

The current systematic review and meta-analysis should be interpreted in the context of its limitations, its conclusions being limited by the volume and quality of the literature available, mostly composed of randomised and cohort studies. In many cases, the studies reviewed were affected by large variability and a lack of high-level evidence. Hence, their retrospective and observational nature makes them susceptible to many sources of bias in the collection and reporting of data, selection of participants and unblinded assessment of outcomes. However, as the funnel plot shows (Figure 3), no specific publication bias was identified. Therefore, we can assume that no relevant elements of heterogeneity are present in the samples included in the meta-analysis that would have influenced the results. We also acknowledge that the number of studies included in the review was too small to obtain a statistically significant comparison of the various prevention strategies to define the best prevention protocol of these injuries. To this aim, we suggest that future high-quality studies should include prevention-free control groups to understand the true effectiveness of every intervention analysed in preventing muscular injuries. Finally, for methodological reasons, it is recommended to always record and report the exposure time to the prevention protocols and to the sport activity, as well as to quantify the number of hours and the rate of injury.

## 5. Conclusions

To the best of our knowledge, this review is the first to date to provide a comprehensive description of the available preventive interventions to reduce the incidence of injury to the hamstring muscles among professional and semi-professional football players, such as eccentric exercise (NHE), FIFA 11+ protocol, balance training exercises and exercises to improve core stability control. Our meta-analysis found an overall effectiveness of all available preventive interventions in reducing the incidence of HSIs of football players. However, due to the different sample sizes of the various interventions and the limited number of articles reported in the literature, it was not possible to clearly define the superiority of one prevention protocol over the others.

## Figures and Tables

**Table 1:** Details of the studies included in the review.

Author (Pubbliction Date)	Whalan et al. (2019)	Elerian et al. (2019)		Espinosa et al. (2015)	Kraemer et al. (2009)	Melegati et al. (2013)	Silvers-Granelli et al. (2015)	Grooms et al. (2013)	Petersen et al. (2011)	Total
**Type of study**	Randomized Clinical Trial	Randomized Clinical Trial		Randomized Clinical Trial	Cohort Study	Case Series	Randomized Clinical Trial	Cohort Study	Randomized Clinical Trial	
**Level of Evidence**	I	I		I	III	IV	I	III	I	
**n(M/F)**										
***IG***	408 (M)	*IG 1:* 17 (M)	*IG 2:* 17 (M)	22 (F)	120 (F)	36 (M)	675 (M)	34 (M)	461 (M)	1648 (M)/142 (F)
***CG***	398 (M)	35 (M)		21 (F)	24 (F)	36 (M)	850 (M)	30 (M)	481 (M)	1830 (M)/45 (F)
AGE * (years)										
***IG***	23.8	24.2 ± 3.2	24.5 ± 4.4	20 ± 3	21.0 ± 4	U/D	20.68 ± 1.46	20.0 ± 2.4	23.5 ± 4	
***CG***	24.8	(same team)		20 ± 3	21.0 ± 4	U/D	20.40 ± 1.66	20.3 ± 1.6	23 ± 4	
**BMI (kg/m^2^)**										
***IG***	U/D	22.4 ± 0.4	22.5 ± 0.2	23.3 ± 1.8	26.9 ± 1.2	U/D	U/D	U/D	U/D	
***CG***	U/D	(same team)		23.3 ± 1.8	26.9 ± 1.2	U/D	U/D	U/D	U/D	

n(M/F) = males/females; IG = intervention group; CG = control group; Age = * average value; same team = the CG is the team from the previous season; U/D = undetermined.

**Table 2:** Quality assessment: scores of the studies included in the review, using the Kennelly Scale [53].

	Melegati et al.	Kraemer et al.	Grooms et al.	Whalan et al.	Elerian et al.	Silvers-Granelli et al.	Petersen et al.	Espinosa et al.
1.	1	1	1	1	1	1	1	1
2.	1	1	1	1	1	1	1	1
3.	1	1	1	1	1	1	1	1
4.	0	0	0	1	1	1	1	1
5.	0	0	0	0	1	1	1	1
6.	1	1	1	1	1	1	1	1
7.	1	1	1	1	1	1	0	0
8.	0	0	0	0	0	0	1	0
9.	1	1	1	1	1	1	1	1
10.	0	1	1	1	1	1	1	1
11.	0	0	0	0	0	0	1	1
12.	U/D	U/D	1	1	1	U/D	1	1
13.	0	1	0	1	0	1	1	1
14.	U/D	U/D	U/D	U/D	1	1	1	1
15.	U/D	U/D	1	0	1	1	1	1
16.	1	1	1	1	1	1	1	1
17.	0	0	1	0	1	1	1	1
18.	0	0	0	U/D	U/D	U/D	1	1
19.	0	1	1	1	U/D	U/D	1	1
20.	1	1	1	1	1	1	1	1
21.	1	1	1	1	1	1	0	0
22.	0	0	0	1	1	1	1	1
23.	0	1	1	1	1	1	1	1
24.	0	1	1	1	1	1	1	1
25.	1	1	1	1	1	1	1	1
26.	1	1	1	1	1	1	0	1
27.	1	1	1	1	1	1	0	1
28.	1	1	1	1	1	1	0	1
29.	0	0	0	1	0	1	1	1
30.	0	0	0	1	1	1	1	1
31.	U/D	U/D	U/D	1	0	U/D	1	1
32.	0	0	0	0	0	0	1	0
33.	U/D	U/D	1	1	1	1	1	1
34.	U/D	U/D	0	0	1	U/D	U/D	1
TOTAL	13/34	18/34	21/34	25/34	26/34	26/34	28/34	30/34
Quality of the study	Poor	Fair	Good	Good	Good	Good	Good	Good
%	38.2	52.9	61.8	73.5	76.5	76.5	82.4	88.2

U/D = undetermined.

**Table 3:** Detailed preventive interventions performed in the studies included in the review.

Author	Groups (=n)	Type of Protocol	EXP. (in h)	HSI (n)	Follow-Up (Weeks)	HI/1.000 h
Whalan et al.	IG (=408)	FIFA 11+ modified	28,541.4	45	28–34	1.58/1000 h
	CG (=398)	FIFA 11+	26,062.1	51	28–34	1.97/1000 h
Elerian et al.	IG (=17)	Nordic Hamstring Exercise (NHE) Protocol pre and post training	1997.1	1	12	0.5/1000 h
	IG 2 (=17)	NHE Protocol pre training	1999.2	4	12	2/1000 h
	CG (=35)	No prevention programme	4095	20	12	4.89/1000 h
Espinosa et al.	IG (=22)	Eccentring Training for 21 weeks, NHE and eccentric band exercise (EBE)	N/D	1	28	U/D
	CG (=21)	Programme with leg swings (FLS), side leg swings (SLS) e multiple jumping (MJ)	N/D	5	28	U/D
Kraemer et al.	IG (=120)	Balance Training Calculated in minutes after training sessions	TOT: 10,079	TOT: 72	TOT: 90–100	7.14/1000 h
	CG (=24)	No prevention programme	2003/2004: 2010	25	18–20	12.44/1000 h
Melegati et al.	IG (=36)	Core Stability Exercises	8041	11	42	1.37/1000 h
	CG (=36)	No prevention programme	4848	11	30	2.27/1000 h
Silvers-Granelli et al.	IG (=675)	FIFA 11+	44,212	16	20–22	0.45/1000 h
	CG (=850)	No prevention programme	35,226	55	20–22	1.24/1000 h
Grooms et al.	IG (=34)	FIFA 11+	2703	1	32–36	0.37/1000 h
	CG (=30)	No prevention programme	2418	5	32–36	2.07/1000 h
Petersen et al.	IG (=461)	NHE Protocol	U/D	15	80	U/D
	CG (=481)	No prevention programme	U/D	52	80	U/D
Total	IG (=1790)		95,093.4	165		
	CG (=1875)		75,128.4	224		
Total of Patients	(=3665)		170,221.8	389		

U/D = undetermined; EXP. = exposure; HI = hamstring injuries; IG = intervention group; CG = control group.

## Data Availability

The dataset generated during the current study is available from the corresponding authors on reasonable request.
